# Association of Procurement Time With Pancreas Transplant Outcomes in Brain-Dead Donors

**DOI:** 10.3389/ti.2023.11332

**Published:** 2023-06-29

**Authors:** Verner Eerola, Ville Sallinen, Marko Lempinen, Ilkka Helanterä

**Affiliations:** Department of Transplantation and Liver Surgery, Helsinki University Hospital and the University of Helsinki, Helsinki, Finland

**Keywords:** graft survival, organ procurement, brain death, pancreas allograft function, early graft loss

## Abstract

A brain-death-induced cytokine storm damages organs in an organ donor. However, a longer time period between declaration of brain death and organ procurement (procurement interval) is associated with improved outcomes in kidney, liver, heart, and lung transplantation. The aim of this study was to find the optimal procurement interval for pancreas transplantation. Association of procurement interval with pancreas graft outcomes was analyzed using multivariable models adjusted for variables possibly affecting procurement interval and outcomes. Altogether 10,119 pancreas transplantations were included from the Scientific Registry of Transplant Recipients. The median follow-up was 3.2 (IQR 1.01–6.50) years. During the first year, 832 (9.0%) grafts were lost, including 555 (6.0%) within the first 30 days. Longer procurement interval was associated with increased death-censored graft survival in a multivariable model (HR 0.944 95% CI 0.917–0.972, per 10-h increase, *p* < 0.001). A decreasing hazard of graft loss was observed also with 1-year, but not with 30-day graft survival. During 1-year follow-up, 953 (12.1%) patients had an acute rejection, and longer procurement interval was also associated with less acute rejections (OR 0.937 95% CI 0.900–0.976, per 10-h increase, *p* = 0.002) in the multivariable model. In conclusion, longer procurement interval is associated with improved long-term outcomes in pancreas transplantation.

## Graphical Abstract

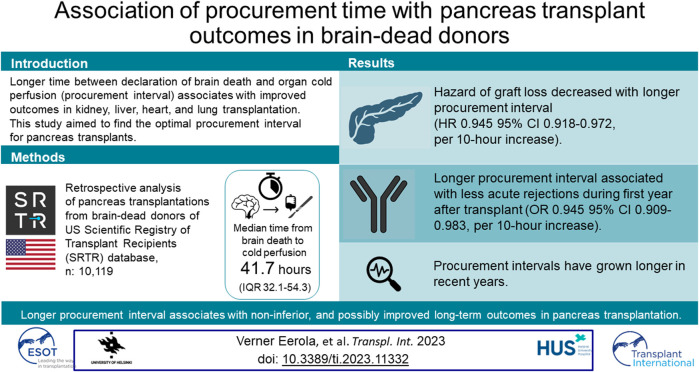

## Introduction

As practically all pancreas allografts are obtained from deceased donors, and as there continues to be hesitation regarding using pancreata from donors after circulatory death, roughly 97% of transplanted pancreata are affected by brain death and the resulting “cytokine storm” [1, 2]. The following hemodynamic instability, possible organ sensitization, and blood coagulation disorders have led to cell damage and ischaemia in various organ systems in animal and human studies [3–6].

To minimize this possible damage, some transplant centers have aimed to minimize time to procurement; although, in recent decades, evidence to support continuous organ injury is sparse, and donor losses from hemodynamic instability are rare [7–9]. Interestingly, a publication in 1994 identified that prolonging procurement interval after donor brain death was associated with increased risk of pancreas graft thrombosis and graft loss [10].

In other retrospective studies of kidney, lung, liver, and heart transplantation, waiting before procurement seems beneficial, at least up to 50 h after brain death [11–18]. Organ reactions to brain death can differ and finding the optimal time for procurement of pancreas grafts could improve transplantation logistics and outcomes as pancreas transplants suffer from the highest incidence of non-immunologic complications of all solid organ transplants—often leading to graft loss [1, 19].

This study aimed to analyze the association of procurement interval (time from declaration of brain death to organ cold perfusion) with pancreas allograft survival and acute rejections in a retrospective cohort from the United States.

## Materials and Methods

This study used data from the Scientific Registry of Transplant Recipients (SRTR). The SRTR data system includes data on all donors, wait-listed candidates, and transplant recipients in the US, submitted by the members of the Organ Procurement and Transplantation Network (OPTN). The Health Resources and Services Administration (HRSA), U.S. Department of Health and Human Services provides oversight to the activities of the OPTN and SRTR contractors.

Pancreas transplantations from brain-dead donors recorded in the SRTR database in the US between January 2010 and September 2021 were included. Follow-up was recovered for all patients from SRTR Standard Analysis Files. Living and donation after circulatory death (DCD) donors were excluded.

This study was approved by the Institutional Review Board of Helsinki University Hospital (HUS/459/2018) and SRTR. The clinical and research activities being reported are consistent with the Principles of the Declaration of Istanbul as outlined in the “Declaration of Istanbul on Organ Trafficking and Transplant Tourism” and the Declaration of Helsinki.

### Variables

Procurement interval was defined as the time between the declaration of brain death and the start of *in situ* cold perfusion. Brain death is generally diagnosed according to strict criteria, which include having a cause of death, testing for the absence of brainstem-, and pain reflexes, and apnea. Additional testing proceeds from uncertainty of any of the above [20].

The following donor data were gathered: the time of declaration of brain death, start time of cold perfusion in organ procurement surgery, location, donor age, gender, cause of death, body mass index and history of resuscitation, inotrope use, hypertension, and diabetes. The obtained recipient and transplantation data included recipient age, gender, body mass index, history of hypertension, human leukocyte antigen mismatches, calculated panel-reactive antibodies (CPRA), time in dialysis, previous transplants, graft cold ischemia time, organ location, acute rejection episodes before discharge from hospital and before follow-ups, and graft survival during the follow-up.

### Endpoints

Death-censored graft survival (measured as centers’ reporting to follow-up forms) was chosen as the primary dependent outcome measure. Secondary endpoints were graft survival at 30 days after the operation, graft survival 1 year after the operation and acute rejections during the first year after transplantation. The definition of pancreas graft failure has evolved from center-specific definitions of either a degree of insulin-independence or C-peptide production, to (from 2018 onwards) a uniform measurement of either: 1. Removal of graft, 2. Patient waitlisted for retransplantation or islet transplantation, 3. Patient death, or 4. Recipients total insulin need is ≥0.5 units/kg/day for 90 consecutive days [1]. This definition has been criticized of having a large cut-off of insulin dosage [21]. In this study a center-specific report of pancreas graft “loss” is accepted as a meaningful endpoint as the cohort is large.

### Statistical Analysis

Characteristics of data in the tables are reported with median and interquartile range (IQR) for continuous data and frequencies with percentages for categorical data. Number of patients with missing values are stated in Table 1. Tertiles of procurement interval were used to divide the data into groups in Table 1 for assessment of uneven distribution of variables and allograft quality.

**TABLE 1:** Characteristics of donors and recipients of pancreas transplants performed between January 2010 and September 2021 in the US and recorded to the SRTR database and divided to tertiles by procurement interval.

Variable	Median and interquartile range or n (valid %). N: 10,119	Missing (%)	1st n: 3,365 (33.33%) (0–35.25 h)	2nd n: 3,387 (33.35%) (35.25–49.37 h)	3rd n: 3,367 (33.33%) (49.37 h->)
			Median (IQR) or n (%)	Median (IQR) or n (%)	Median (IQR) or n (%)
Donor Age, years	23 (18–29)	0 (0%)	23 (18–30)	23 (18–30)	23 (19–29)
Donor BMI, kg/m^2^	23.6 (21.2–26.2)	0 (0%)	23.7 (21.3–26.3)	23.5 (21.1–26.0)	23.7 (21.0–26.3)
Donor, male	7,006 (69.2%)	0 (0%)	2,300 (68.4%)	2,369 (69.9%)	2,337 (69.4%)
Donor Hypertension	448 (4.4%)	43 (0.4%)	156 (4.6%)	160 (4.7%)	132 (3.9%)
Donor Cause of Death, stroke	1,215 (12.0%)	0 (0%)	443 (13.2%)	388 (11.5%)	384 (11.4%)
Donor organ yield^a^	5 (5–6)	0 (0%)	5 (5–6)	5 (5–6)	5 (5–6)
Local donor	7,157 (70.7%)	0 (0%)	2,450 (72.8%)	2,377 (70.2%)	2,330 (69.2%)
Machine perfusion used for SPK kidneys	1,235 (15.4%)	3 (0.0%)	451 (17.3%)	381 (14.1%)	403 (14.7%)
Inotrope use for donor	4,687 (46.3%)	15(0.1%)	1,835 (54.5%)	1,556 (45.9%)	1,296 (38.5%)
≥3 inotropes during procurement	65 (0.6%)	5,665 (56.0%)	40 (1.7%)	19 (1.3%)	6 (0.8%)
Recipient Age, years	42 (35–49)	0 (0%)	42 (35–49)	42 (35–49)	42 (35–49)
Recipient BMI, kg/m^2^	25.3 (22.7–28.2)	82 (0.8%)	25.3 (22.6–28.2)	25.2 (22.7–28.0)	25.4 (22.7–28.3)
Recipient Gender, male	5,964 (58.9%)	0 (0%)	1,984 (59.0%)	2,006 (59.2%)	1,974 (58.6%)
Recipient Hypertension	3,554 (81.6%)	5,762 (56.9%)	1,654 (80.6%)	1,153 (82.2%)	747 (82.7%)
Previous Transplants	1,316 (13.0%)	0 (0.0%)	528 (15.7%)	429 (12.7%)	359 (10.7%)
HLA^b^ mismatches	5 (4–5)	5 (0%)	5 (4–5)	5 (4–5)	5 (4–5)
Recipient CPRA^c^ >20%	1,911 (18.9%)	5 (0%)	599 (17.8%)	642 (19.0%)	670 (19.9%)
Transplant type^d^,				
SPK	8,046 (79.5%)	0 (0%)	2,600 (77.3%)	2,700 (79.7%)	2,746 (81.6%)
PAK	970 (9.6%)	0 (0%)	407 (12.1%)	300 (8.9%)	263 (7.8%)
PTA	1,103 (10.9%)	0 (0%)	358 (10.6%)	387 (11.4%)	358 (10.6%)
Follow-up Time, years	3.2 (1.0–6.5)	0 (0%)	5.1 (1.9–8.0)	3.4 (1.1–6.0)	2.0 (0.8–4.3)
Transplant year	2015 (2012–2018)	0 (0%)	2013 (2011–2016)	2016 (2013–2018)	2018 (2015–2020)

^a^ Number of organs donated (kidney, kidney, liver, pancreas, intestine, lungs, heart).

^b^ Human leukocyte antigen.

^c^ Calculated panel-reactive antibodies.

^d^ SPK, simultaneous pancreas and kidney transplantation; PAK, pancreas after kidney; PTA, pancreas transplant alone.

**FIGURE 1:**
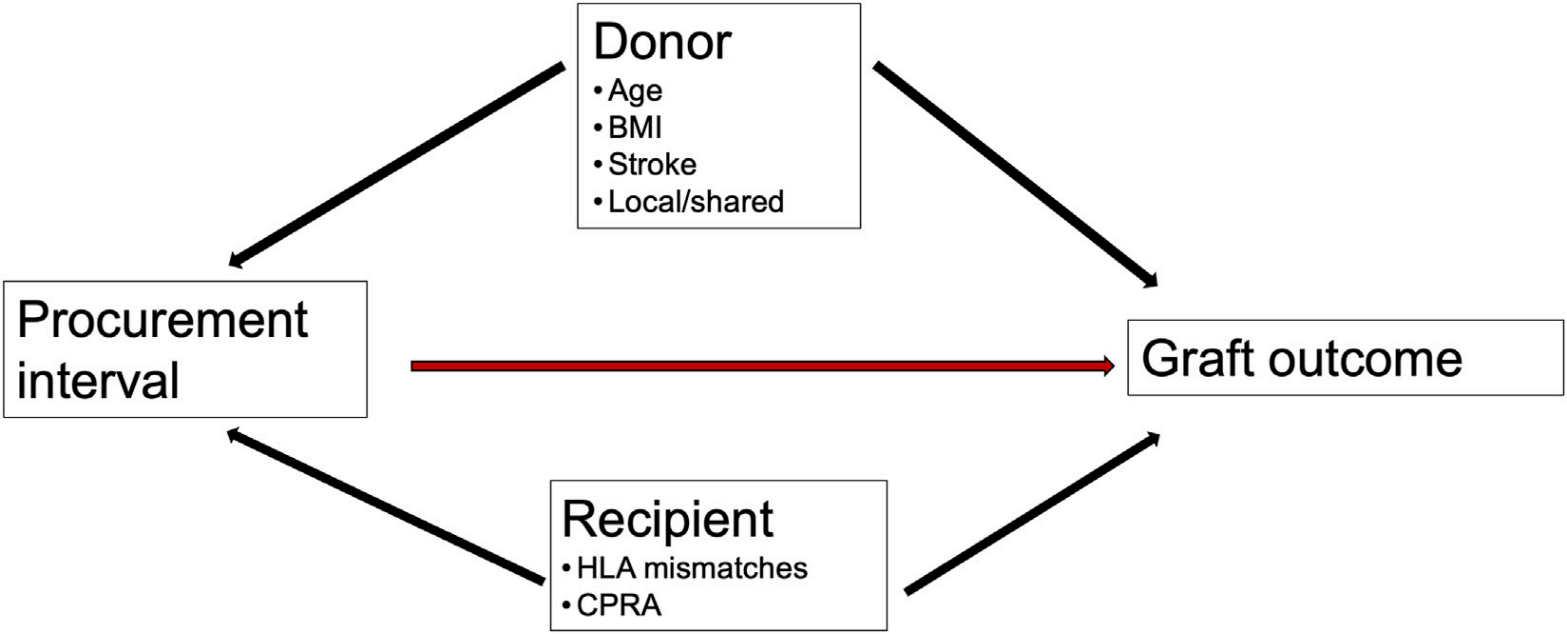
Directed acyclic graph. Confounders of association of procurement interval (time from brain death to organ cold perfusion) with outcomes.

Confounder analysis for the multivariable model was constructed as a directed acyclic graph (DAG) [22]. The DAG (Figure 1) presents our team’s theory of factors possibly affecting procurement intervals and confounding the associations. Cox proportional hazards models and logistic regression were used for analysis of the association of procurement interval with endpoints and for covariate adjustment. Donor age, donor BMI, donor location as local or shared, stroke as cause of donor death, and recipient’s HLA mismatches, retransplantation, and CPRA were identified as confounders. The very few missing variables were estimated to be randomly distributed allowing complete-case analyses.

The associations were modelled as both linear and non-linear. Potentially non-linear associations were checked for by using restricted cubic spline functions between procurement interval and endpoints, as logistic and Cox regression models require the assumption of linearity for continuous data. The associations are modelled in the figures as non-linear for realization of confidence intervals and data visualization. Linear associations were reported using hazard ratio (HR) and odds ratio (OR) with a 95% confidence interval (CI), and significance of non-linearity was reported with *p*-values. All associations were linear in the final results.

Bias was addressed by the DAG, including all transplantations, testing endpoints for non-linearity, confounder adjustment, and sensitivity analyses for including only SPK recipients and for comparing different eras.

The significance level was set at 5% and analyses were carried out as two-tailed. Analyses were performed using R software, utilizing survival and rms packages (R Foundation for Statistical Computing, Vienna, Austria).

## Results

### Patients

From 2010 to September 2021, 11,919 pancreas transplantations were performed and recorded to the SRTR database in the United States. The final cohort of complete cases included 8,046 simultaneous pancreas and kidney (SPK) transplantations and 2,073 pancreas transplant alone (PTA) or pancreas after kidney (PAK) transplantations, as 311 DCD, 57 transplantations with unreliable procurement interval (>120 h), 449 pediatric recipient transplantations and 983 transplantations with missing brain death time, survival status or follow-up time were excluded.

**FIGURE 2:**
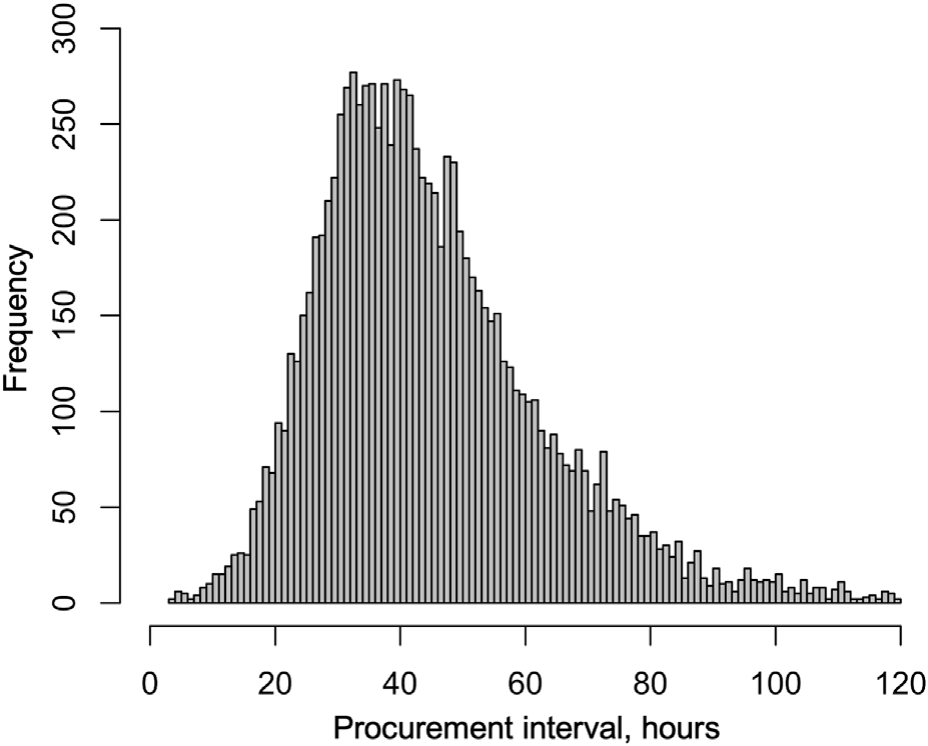
Distribution of procurement intervals in brain-dead donors in the US from January 2010 to September 2021.

Median procurement interval was 41.7 h (IQR 32.1–54.3). The distribution is provided in Figure 2. Table 1 summarizes the characteristics of donors, transplantations, patients, endpoints, and missing values. For description of data, the characteristics are divided by procurement interval tertiles into Table 1 and outcomes into Table 2.

**TABLE 2:** Outcomes of pancreas transplants performed between January 2010 and September 2021 in the US and recorded to the SRTR database, divided to tertiles by procurement interval.

Outcome	Median and interquartile range or n (valid %). *N*: 10,119	Missing (%)	1st *n*: 3,365 (33.33%) (0–35.25 h)	2nd *n*: 3,387 (33.35%) (35.25–49.37 h)	3rd *n*: 3,367 (33.33%) (49.37 h->)
			Median (IQR) or n (%)	Median (IQR) or n (%)	Median (IQR) or n (%)
Graft Loss^a^ <30 days^b^	5.9%	0%	6.4%	5.8%	5.4%
Graft Loss^a^ <1 year^b^	9.1%	0%	10.2%	9.0%	8.0%
Acute Rejection, Before Discharge	164 (1.6%)	14 (0.1%)	63 (1.9%)	53 (1.6%)	48 (1.4%)
Acute Rejection, First Year^c^	953 (12.1%)	1,388 (15.0%)	368 (13.3%)	329 (12.2%)	256 (10.5%)

^a^ Death-censored graft survival defined as center reporting to follow-up form.

^b^ Kaplan-Meier estimated survival percentages with standard errors of 0.004 (30-day)-0.005 (1-year).

^c^ Of 9,280 cases with at least 1 year of follow-up.

### Graft Survival

**FIGURE 3:**
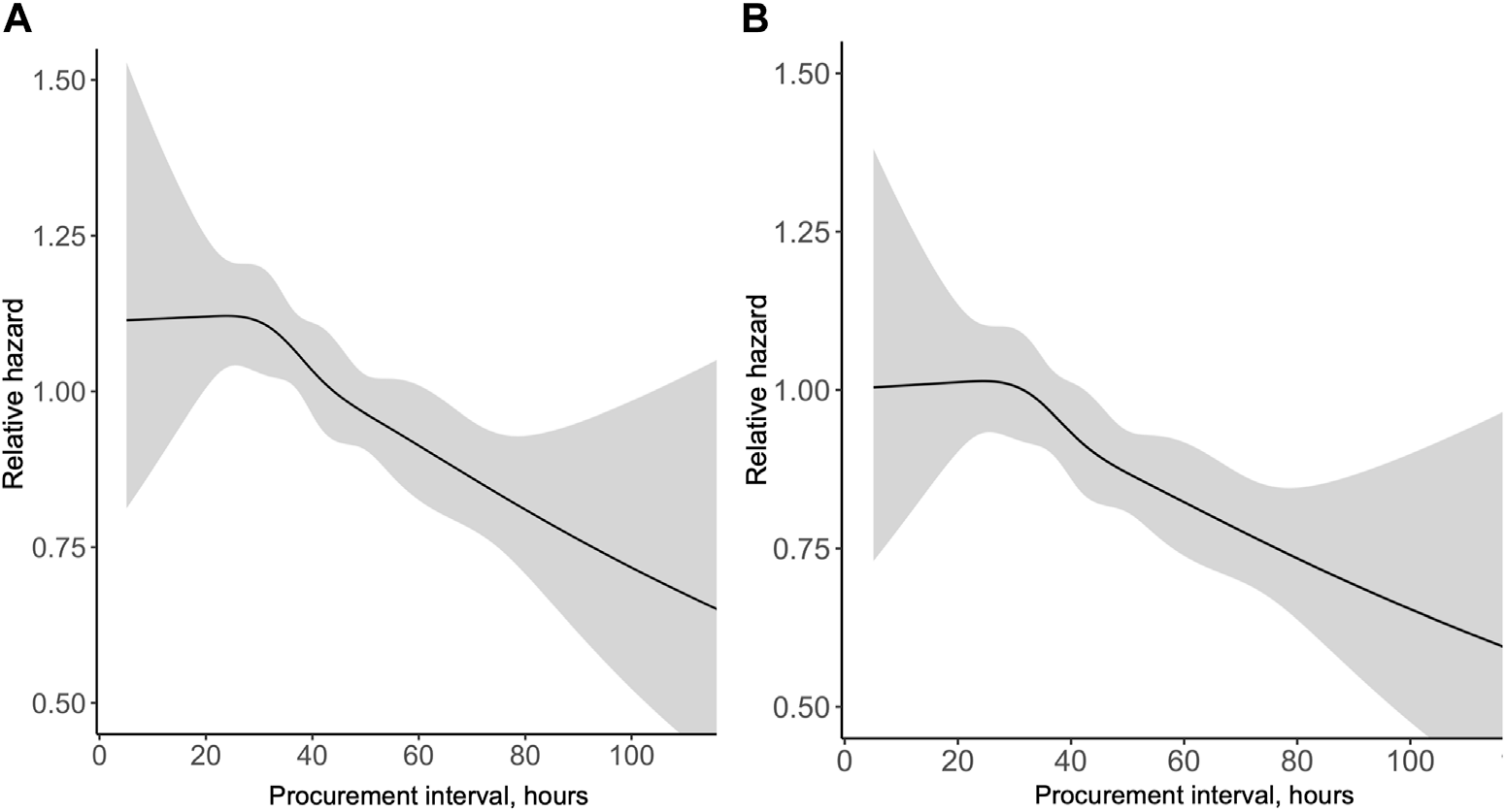
Univariable **(A)** and multivariable **(B)** association of procurement interval with relative hazard of pancreas graft loss [**(A)** Relative to median, **(B)** Relative to minimum interval].

**FIGURE 4:**
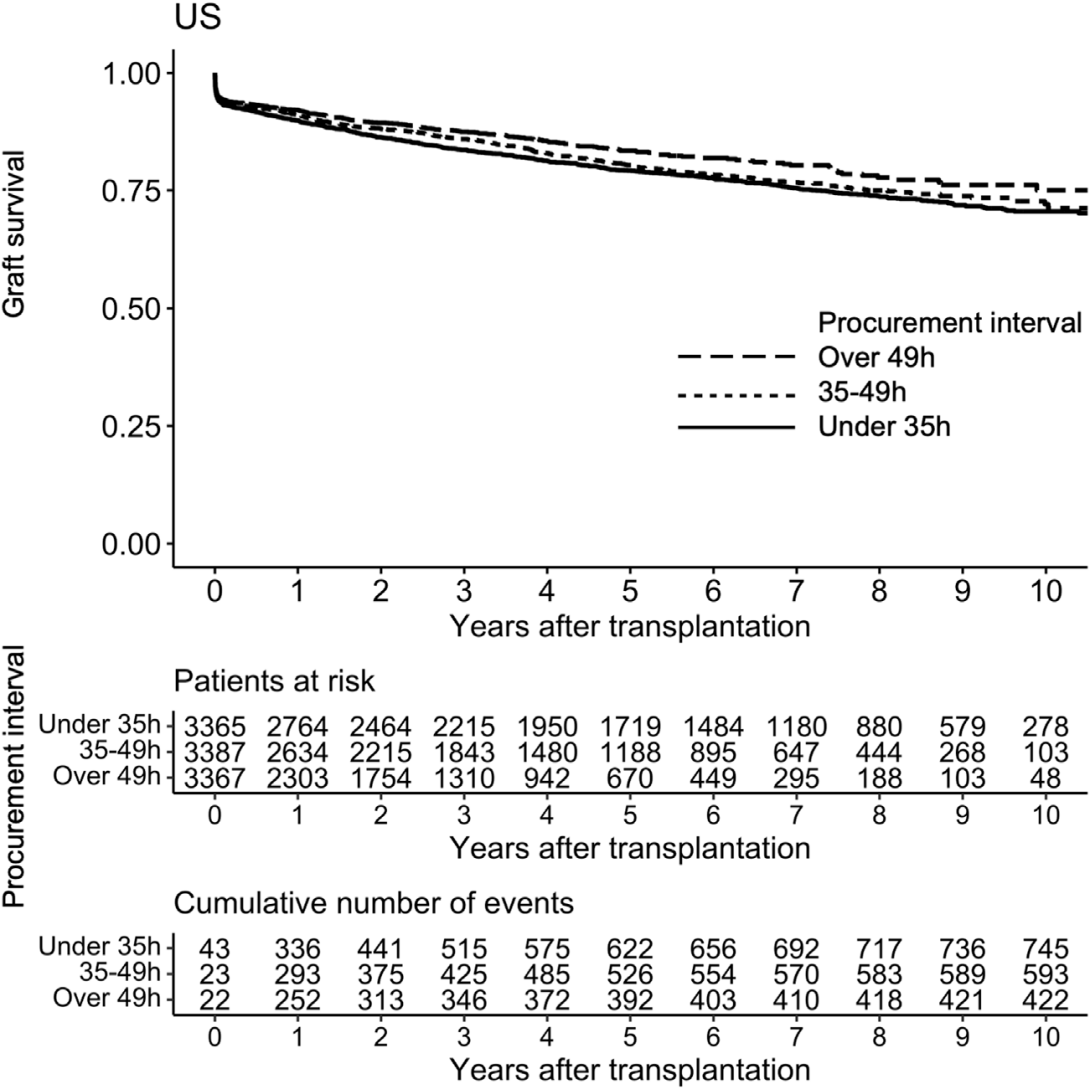
Kaplan-Meier curve of graft survival of pancreas transplant tertiles of procurement interval (time between brain death and cold perfusion).

During the median follow-up period of 3.2 years, 1,764 (17.4%) grafts were lost and 986 (9.7%) patients died. Altogether 593 (5.9%) were lost during the first 30 days and 832 (9.0%) during the first year. In a univariable Cox regression model the association of procurement interval with death-censored graft survival was linear (non-linearity *p* = 0.88, Figure 3) and significant (HR 0.944 95% CI 0.917–0.972 per 10-h increase, *p* < 0.001). The association remained independent in the adjusted model (HR 0.944 95% CI 0.917–0.972 per 10-h increase, *p* < 0.001). For graphical purposes in the Kaplan-Meier curve (Figure 4) the cohort was divided into tertiles for unadjusted interpretation of survival.

In the adjusted model, longer procurement interval was associated with better 1-year graft survival (HR 0.923 95% CI 0.885–0.962 per 10-h increase, *p* < 0.001), but 30-day graft survival was not associated with procurement interval (HR 0.964 95% CI 0.920–1.009 per 10-h increase, *p* = 0.118). As procurement intervals grew longer with time in the cohort, transplant year was added to the 1-year adjusted model as a confounder. In this model, transplant year and the interaction between procurement interval and transplant year were significant, and a longer procurement interval remained significantly associated with improved 1-year graft survival (HR 0.952 95%CI 0.911–0.995 per 10-h increase, *p* = 0.030).

A composite endpoint of graft and patient survival was associated with procurement interval, similarly to death-censored graft survival (Supplementary Tables S2, S3).

### Acute Rejections

**FIGURE 5:**
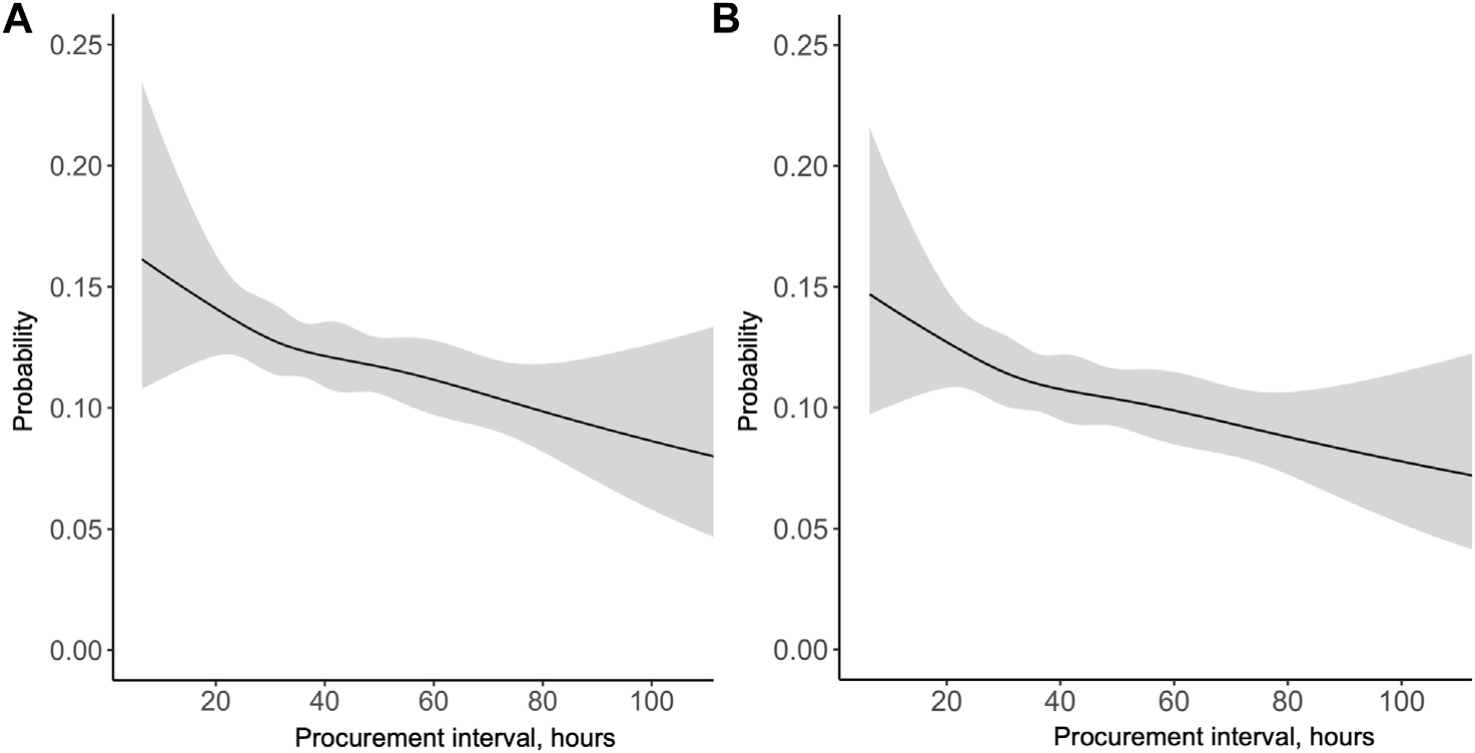
Probability of acute rejection in the first year after pancreas transplantation by procurement interval in the **(A)** univariable and **(B)** multivariable model.

During the study period, 953 (12.1%) patients had an acute rejection episode before 1-year of follow-up. In a univariable logistic regression model the association of procurement interval with acute rejection within 1 year was significant (OR 0.938 95% CI 0.901–0.977 per 10-h increase, *p* = 0.002) and linear (non-linearity *p* = 0.96, Figure 5). When adjusted, longer procurement interval was associated with less acute rejections within 1 year (OR 0.937 95% CI 0.900–0.976 per 10-h increase, *p* = 0.002, Supplementary Figure S1).

### Sensitivity Analyses

#### Simultaneous Pancreas and Kidney (SPK) Transplantations

**FIGURE 6:**
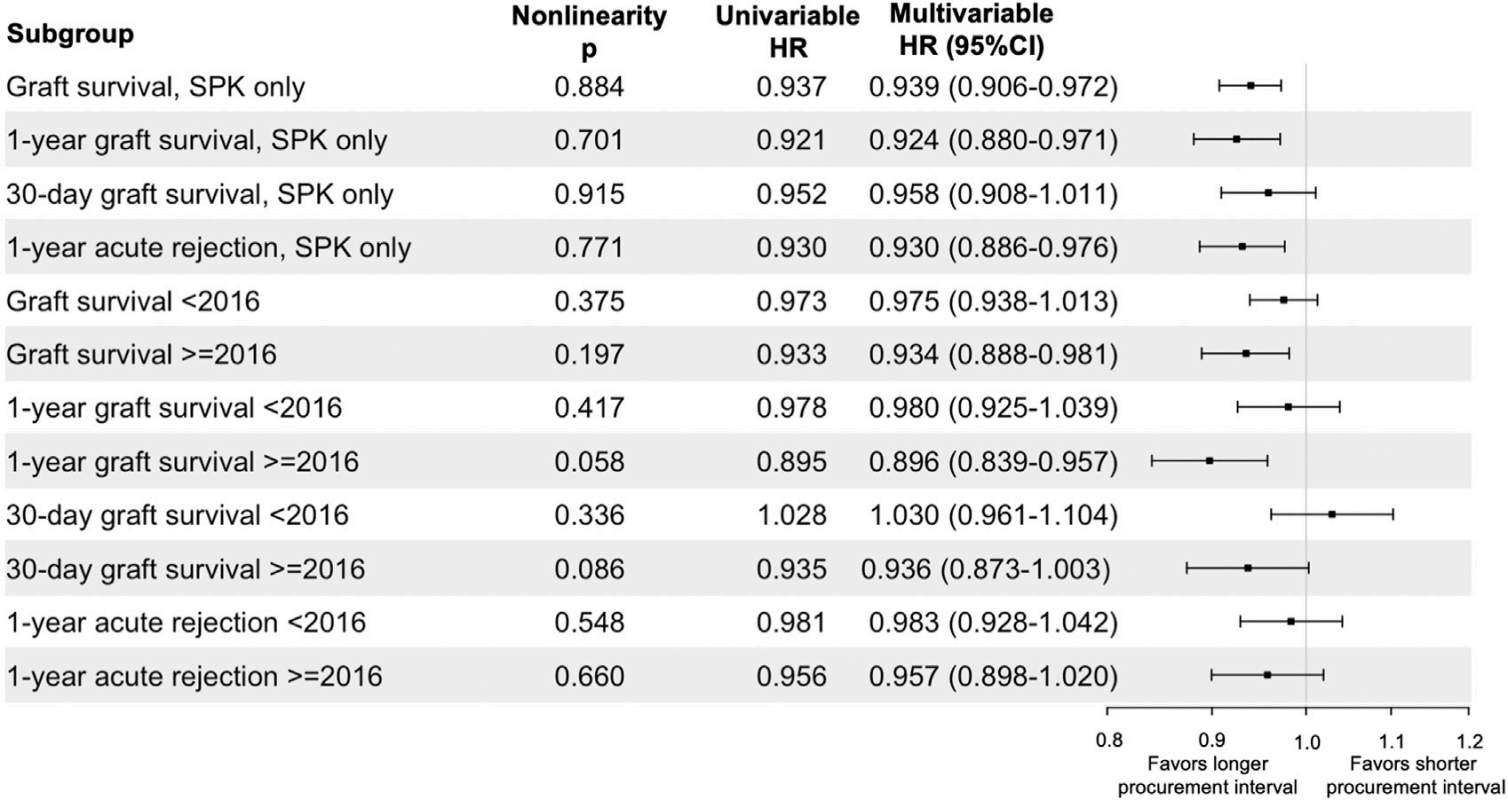
Sensitivity analyses of procurement interval’s association per 10-h increase with endpoints in subgroups. Univariable confidence intervals provided in Supplementary Table S1.

When only SPK transplantations were included, the analyzed associations remained equally significant as in the full cohort during different follow-up periods for graft survival and acute rejections (Figure 6). Procurement interval was beneficially associated with 1-year graft survival of SPK-kidneys in the adjusted analyses (HR 0.897 95% CI 0.814–0.989 per 10-h increase, *p* = 0.029), but not significantly associated with delayed graft function (HR 1.019 95% CI 0.970–1.069 per 10-h increase, *p* = 0.460, Supplementary Tables S2, S3).

#### Transplant Year

Procurement intervals grew longer during the cohort period (Table 1) and thus the cohort was divided in two for an additional sensitivity analysis. Pre-2016 group had 5,115 patients and the post-2016 group included 5,004 patients. The associations of procurement interval with pancreas graft survival in these sub-groups are summarised in Figure 6. The association of procurement interval with acute rejections within 1 year was nonsignificant for both time periods when the cohort was divided by 2016 (Figure 6).

#### Inotropes

Donor inotrope use at the start of the procurement operation decreased with longer procurement interval (Table 1). Inotrope use was available for 99.8% of the cohort, and was considered on its own (as a surrogate for donor instability) and with procurement interval. In Cox regression univariable analyses, inotrope use was not significantly associated with graft survival (HR 1.070 95% CI 0.972–1.175 per 10-h increase, *p* = 0.168) or 1-year graft survival (HR 1.109 95% CI 0.968–1.271 per 10-h increase, *p* = 0.137). For 30-day graft survival the association was slightly significant (HR 1.268 95% CI 1.073–1.499 per 10-h increase, *p* = 0.005). When the association of procurement interval with outcomes was adjusted with inotrope use the association of procurement interval remained significant (i.e., for 1-year graft survival HR 0.925 95% CI 0.887–0.964 per 10-h increase, *p* < 0.001). The association also remained significant when the interaction between procurement interval and inotrope use was considered (for 1-year graft survival HR 0.873 95% CI 0.822–0.927 per 10-h increase, *p* < 0.001). The association of procurement interval with 30-day graft survival became significant when inotrope use and the interaction with procurement interval were analyzed (adjusted model HR 0.922 95% CI 0.857–0.991 per 10-h increase, *p* = 0.028).

## Discussion

In this study, longer procurement interval was associated with improved long-term pancreas graft survival and fewer rejections within 1 year. Most importantly, a longer procurement interval posed no additional risk.

Potential donors are seldom lost to cardiovascular collapse, possibly due to improved donor management protocols [7–9]. Earlier dogma of fast procurement might have been due to “unstable” donors with undoubtedly worse outcomes when organ perfusion has been compromised. In earlier settings, longer time after brain death may indeed have posed a risk for transplant. However, it has been proposed that if organ perfusion is kept stable, the organs can recover from the first hit of brain death, and are better prepared for cold ischemia (i.e., the two-hit theory first suggested by Kunzendorf et al [13]) as the autonomous and cytokine storm seems to “cool down” in the hours following brain death [23–25].

This is outlined in recent retrospective studies which point to benefit in outcomes from longer procurement intervals in kidneys, livers, hearts, or lungs [11–18]. This study is in concordance with these studies. The slightly improved graft survival associated with longer procurement intervals could reflect the two-hit theory. Other factors possibly outlining this suggested improvement in graft survival could be related to preconditioning initiated by cytokines that activate expression of protective genes upon brain death [26–30]. Earlier studies may have been confounded by fewer multiorgan-donors pooling into shorter brain death times as time-consuming donor testing non-randomly distributes healthier donors (with better outcomes) into longer intervals. This study shows that the phenomenon seems to exist in the healthiest organ donors—typically multiorgan pancreas donors—as well.

Donor inotrope use decreased with procurement interval, which could be expected with cytokine storm cooling and stabilization of the donor. It could also serve as a surrogate marker for instability. Interestingly, in sensitivity analyses the use of inotropes in the management of the organ donor was not significantly associated with pancreas graft outcomes. However, dichotomous inotrope use before procurement operation is probably insufficient as a marker for donor instability. Unfortunately, the use of three or more inotropes was only reported for a few patients.

Acute rejections have not been associated with procurement times in other organs in earlier studies. The reason for this discrepancy remains unclear, but may on one hand be related to different granularity of reporting acute rejections to large registries, and on the other hand relate to variable sensitization of different organs during the process of brain death [31].

Procurement intervals grew longer during the period of the study cohort, and possibly better donor treatment practices during the later years are associated with the better outcomes of longer procurement intervals. Therefore, sensitivity analyses by transplantation year were conducted, which showed the association of a longer procurement interval with improved graft survival to be significant only in recent years, and the association of less acute rejections to dissipate.

The difference in procurement intervals between Europe and the US is notable, which may arise from scheduling procurement during office hours and more time consuming consent obtainment in the United States (Nijboer). Obviously much less concern about longer procurement intervals exists in the United States.

This study is an observational registry analysis and therefore cannot prove causality. Retrospective studies can be susceptible to non-random allocation and confounding, and residual bias, which cannot be completely overcome by multivariable adjustment or sensitivity analyses. However, the use of the multivariable model and non-linear associations, together with the sensitivity analyses, provide greater confidence in the conclusion. The start of the procurement interval was defined by the declaration of brain death, although the exact time or progression of the fatal brain insult cannot be known. Similarly, the urgency of obtaining the diagnosis of brain death and declaration time and thus the start of the interval can vary between different centers and practises. However, this variance should be balanced in a large cohort. Graft failure for pancreas allografts has been uniformly defined in the US only from 2020 onwards, which can also have an influence on our findings. A lack of definition for pancreas graft function could have resulted in variation in reporting complications. Still, a large cohort can alleviate many of these concerns.

A possible selection bias follows from longer procurement times distributing to more recent years. We sought to limit this with sensitivity analyses, which do not point to the effect resulting from better care in the later years, but did show significantly better results with a longer procurement interval only for later years in terms of graft survival, and no significance for acute rejections. Also, other short-term complications, such as graft thrombosis and leaks would have been of interest in this study, but are unfortunately outside the scope of the used registry data.

A concern in optimizing other organs by lengthening the procurement interval is that more pancreas grafts would become edematous or firm and lead to more discard of the pancreas grafts. Before suggesting delaying procurement, information on discard rates with a longer procurement interval would be insightful.

Future studies with randomization by procurement interval would be welcomed but could prove ethically challenging and unwarranted since so many other factors weigh first in organ procurement. Also, studies on how many donors are lost to cardiovascular collapse during the current era of donor management protocols would be of interest. These studies would have clinical implications for transplantation logistics and patient outcomes.

In conclusion, based on this study, pancreas procurement from a brain-dead donor can be postponed if needed, and a longer procurement interval may lead to better outcomes.

## Data Availability

The datasets presented in this article are not readily available because Restrictions apply to the availability of the data based on the current data use agreements with SRTR. Other data are available from the corresponding author upon reasonable request. Requests to access the datasets should be directed to SRTR, srtr@srtr.org.

## Abbreviations

CPRA, calculated panel-reactive antibodies; DAG, directed acyclic graph; HR, hazard ratio; IQR, interquartile range; OPTN, organ procurement and transplantation network; OR, odds ratio; PAK, pancreas after kidney transplantation; PTA, pancreas transplant alone; SPK, simultaneous pancreas and kidney transplantation; SRTR, scientific registry of transplant recipients.
