# Supplementary material for: Association of Procurement Time With Pancreas Transplant Outcomes in Brain-Dead Donors
Source: Transpl Int. 2023 Jun 29;36:11332. doi: 10.3389/ti.2023.11332 (PMC10353260; doi:10.3389/ti.2023.11332)
Supplement: Supplementary file 1 [file DataSheet1.docx]

**Supplemental material**

Table of Contents

[Supplemental Table 1. 1](#_Toc134898412)

[Supplemental Table 2. 1](#_Toc134898413)

[Supplemental Table 3. 2](#_Toc134898414)

[Supplemental Figure 1. 3](#_Toc134898415)

[Supplemental Figure 2. 3](#_Toc134898416)

**Supplemental Table 1.** Sensitivity analyses of procurement interval’s association per 10-hour increase with hypothesized endpoints in subgroups. Univariable hazard ratios included.

| Association with procurement interval | Nonlinearity | Univariable^1^ HR (95% CI) | Adjusted^1^ HR (95% CI) |
| --- | --- | --- | --- |
| Full follow-up graft survival, SPK only | P=0.884 | **0.937 (0.905-0.970) p=<0.001** | **0.939 (0.906-0.972) p=<0.001** |
| 1-year graft survival, SPK only | P=0.701 | **0.921 (0.877-0.967) p=0.001** | **0.924 (0.880-0.971) p=0.002** |
| 30-day graft survival, SPK only | P=0.915 | 0.952 (0.902-1.005) p=0.074 | 0.958 (0.908-1.011) p=0.120 |
| 1-year acute rejection, SPK only | P=0.771 | **0.930 (0.886-0.976) p=0.003** | **0.930 (0.886-0.976) p=0.003** |
| Graft survival  <2016  >=2016 | P=0.375  P=0.197 | 0.973 (0.937-1.011) p=0.162  **0.933 (0.888-0.981) p=0.006** | 0.975 (0.938-1.013) p=0.190  **0.934 (0.888-0.981) p=0.007** |
| 1-year graft survival  <2016  >=2016 | P=0.417  P=0.058 | 0.978 (0.922-1.036) p=0.445  **0.895 (0.838-0.956) p<0.001** | 0.980 (0.925-1.039) p=0.501  **0.896 (0.839-0.957) p=0.001** |
| 30-day graft survival  <2016  >=2016 | P=0.336  P=0.086 | 1.028 (0.959-1.101) p=0.440  0.935 (0.872-1.002) p=0.056 | 1.030 (0.961-1.104) p=0.401  0.936 (0.873-1.003) p=0.061 |
| 1-year acute rejection  <2016  >=2016 | P=0.548  P=0.660 | 0.981 (0.926-1.039) p=0.518  0.956 (0.897-1.019) p=0.167 | 0.983 (0.928-1.042) p=0.572  0.957 (0.898-1.020) p=0.180 |
| ^1^Per 10-hour increase | | | |

**Supplemental Table 2.** Sensitivity analyses of procurement interval’s association per 10-hour increase with additional endpoints.

| Association with procurement interval | Nonlinearity | Univariable^1^ HR (95% CI) | Adjusted^1^ HR (95% CI) |
| --- | --- | --- | --- |
| Full follow-up, composite (of graft and patient) survival | P=0.494 | **0.963 (0.940-0.986) p= 0.002** | **0.964 (0.942-0.988) p=0.003** |
| 1-year composite survival | P=0.260 | **0.927 (0.895-0.961) p<0.001** | **0.930 (0.897-0.963) p<0.001** |
| 30-day composite survival | P=0.425 | 0.962 (0.919-1.007) p=0.096 | 0.965 (0.922-1.010) p=0.121 |
| SPK Kidney survival | P=0.339 | 0.959 (0.918-1.002) p=0.062 | 0.961 (0.919-1.004) p=0.072 |
| SPK Kidney 1-year survival | P=0.226 | **0.898 (0.815-0.989) p=0.029** | **0.897 (0.814-0.989) p=0.029** |
| SKP Kidney DGF | P=0.255 | 1.018 (0.970-1.068) p=0.476 | 1.019 (0.970-1.069) p=0.460 |

**Supplemental Table 3.** Additional outcomes of pancreas and pancreas-kidney transplants performed between January 2010 and September 2021 in the US and recorded to the SRTR database, divided to tertiles by procurement interval.

| **Outcome** | **Median and interquartile range or n (valid %). N: 10,119** | **Missing (%)** | **1^st^ n:3,365 (33.33%) (0-35.25h)**  **Median (IQR) or n (%)** | **2^nd^ n:3,387 (33.35%) (35.25-49.37h)**  **Median (IQR) or n (%)** | **3^rd^ n:3,367 (33.33%) (49.37h->)**  **Median (IQR) or n (%)** |
| --- | --- | --- | --- | --- | --- |
| 10-year composite survival^\|\|^ | 58.5% | 0% | 56.8% | 58.5% | 59.8% |
| 1-year composite survival^\|\|#^ | 87.7% | 0% | 86.6% | 87.5% | 89.3% |
| 30-day composite survival^\|\|^ | 93.8% | 0% | 93.1% | 93.9% | 94.3% |
| SPK Kidney 10-year graft survival^¶^ | 76.4% | 0% | 75.6% | 75.7% | 77.6% |
| SPK Kidney 1-year survival^#¶^ | 97.7% | 0% | 97.6% | 97.3% | 98.2% |
| SKP Kidney delayed graft function | 615 (7.6) | 12 (0.1%) | 197 (7.6%) | 185 (6.9%) | 233 (8.5%) |

|| Composite outcome of death and graft survival defined as center reporting to follow-up form, Kaplan-Meier estimate; # Of 9,280 cases with at least one year of follow-up; ¶ Death-censored graft survival, Kaplan-Meier estimate of 8046 SPK transplantations

**Supplemental Figure 1.** Occurrence of acute rejections first year after transplantation (bars) by procurement interval (distribution of cases in percentages for clarity).

**Supplemental Figure 2.** Kaplan-Meier curve of graft survival of pancreas transplants divided to two groups by procurement interval (time between brain death and cold perfusion).
